# Outpatient Management of Pulmonary Embolism Patients with Direct Oral Anticoagulants: A Systematic Review

**DOI:** 10.3390/jcm14248931

**Published:** 2025-12-17

**Authors:** Alvina S. Khachatryan, Denis V. Rylnikov, Sevara A. Mirakhmedova, Evgeny I. Seliverstov, Evgeny S. An, Igor A. Zolotukhin

**Affiliations:** Department of Fundamental and Applied Research in Cardiovascular Surgery, Pirogov Russian National Research Medical University, Moscow 117997, Russia; alvina.khachatryan@yandex.ru (A.S.K.); rylnikov@yahoo.com (D.V.R.); kelly.gellespy@gmail.com (S.A.M.); flebolog@rambler.ru (E.I.S.);

**Keywords:** pulmonary embolism, DOAC, outpatient treatment, apixaban, rivaroxaban

## Abstract

**Background:** Pulmonary embolism (PE) patients are generally admitted to the hospital, which leads to significant burden on healthcare systems. Many PE patients are hemodynamically stable and can be ambulated safely. Direct oral anticoagulants (DOACs) do not need routine laboratory monitoring, which makes them a convenient option for outpatient care. Data on the outpatient management of PE patients using DOACs are scarce. **Methods:** We conducted a systematic search of MECENTRAL databases to identify randomized controlled trials (RCTs), non-randomized studies (non-RCTs), retrospective studies, reviews, systematic reviews, meta-analyses, and clinical guidelines evaluating the use of DOACs for outpatient treatment of PE patients. **Results:** A total of 833 publications were identified, and eight studies—two RCTs and six non-RCTs (two retrospective and four prospective)—were finally included. These studies reported rates of bleeding events, recurrent venous thromboembolism (VTE), PE-related mortality, and all-cause mortality among outpatients who received DOACs. No PE-related deaths were reported in any of the included studies. Bleeding events were reported in five studies, and recurrent VTE rates were reported in two studies. Study findings showed that the sPESI score has some disadvantages in selecting patients for home treatment. The quality of the studies varied considerably. A meta-analysis could not be performed due to heterogeneity in the study designs, patient populations, and outcome reporting. While the treatment results favored outpatient management of selected PE patients, no robust conclusions could be drawn from the published data. **Conclusions:** Outpatient treatment of PE with DOACs has some potential as an approach for managing selected patients. Hestia, POMPE-C and RESI scores are preferable for selecting PE patients for home treatment, but utilizing sPESI as the only tool has to be avoided. Apixaban and rivaroxaban are both safe, with a low bleeding risk. Large-scale prospective studies are required to establish the efficacy and safety of outpatient PE management using DOACs.

## 1. Introduction

Pulmonary embolism (PE) is a life-threatening and burdensome condition. PE is one of the leading causes of cardiovascular mortality [1,2], and PE patients are generally admitted to the hospital. This leads to significant expenses for public healthcare systems, increased risks of in-hospital complications [3,4], and reductions in patients’ mobility and social functioning. Current guidelines for PE indicate outpatient treatment in patients with a low mortality risk [5]. Data from published studies confirm that 30–55% of low-risk PE patients can be discharged after a short-term hospital stay (<24 h) or can be managed as outpatients. Concurrently, most PE patients—including those at low risk of death—are still managed in a hospital setting in real practice [6,7].

Physicians often justify such an approach according to the risk of worsening a patient’s condition and the need for parenteral anticoagulation. After DOACs were proven to be a safe and effective alternative to the previous standard of care—i.e., low-molecular-weight heparins followed by a vitamin K antagonist [8]—outpatient management of PE patients became a matter of discussion. Therapy with DOACs does not require routine laboratory monitoring, which makes these drugs attractive for outpatient PE management. At least hemodynamically stable patients with non-massive PE may benefit from this approach [9].

This study aimed to assess the effectiveness and safety of the outpatient management of PE patients with DOACs based on a systematic analysis of published data.

## 2. Methods

### 2.1. Search Strategy

The Preferred Reporting Items for Systematic Reviews and Meta-Analyses (PRISMA) guidelines were used for reporting the results of this systematic review (Supplementary Materials, Tables S1 and S2) [10]. A literature search across the Cochrane Central Register of Controlled Trials (CENTRAL) and PubMed (MEDLINE) databases was conducted to identify randomized controlled trials (RCTs), non-RCTs, retrospective studies, reviews, systematic reviews, meta-analyses, and clinical guidelines investigating the outpatient treatment of PE patients with DOACs. Additionally, reference lists of all identified and related publications were screened to identify relevant studies. The search was conducted from May 2024 to August 2024.

The search strategy used for the CENTRAL database was as follows:

#1   direct oral anticoagulant;
#2   direct oral anticoagulants;
#3   #1 or #2;
#4   “PE”;
#5   pulmonary embolism;
#6   #4 or #5;
#7   ambulatory;
#8   outpatient;
#9   home;
#10 treatment;
#11 #7 or #8 or #9;
#12 #11 and #10;
#13 rivaroxaban;
#14 apixaban;
#15 dabigatran;
#16 edoxaban;
#17 #13 or #14 or #15 or #16;
#18 ((#3 or #17) and #6) and #11.

The search strategy used for the PubMed database was as follows:

(“pulmonary embolism”) AND (“outpatient treatment” OR “home treatment” OR “ambulatory care”) AND (“dabigatran” OR “rivaroxaban” OR “apixaban” OR “edoxaban” OR “DOAC” OR “direct oral anticoagulants”);

(“pulmonary embolism”) AND (“outpatient treatment” OR “ home treatment” OR “ambulatory care”) AND (“ DOAC” OR “oral anticoagulants”);

(“pulmonary embolism”) AND (“outpatient treatment” OR “ home treatment” OR “ambulatory care”).

The filters used included clinical trial, meta-analysis, review, systematic review, and randomized clinical trial.

Studies were considered eligible if they reported efficacy and safety endpoints, clear patient selection criteria, detailed descriptions of interventions, and the duration of follow-up. Only papers published in English were assessed for eligibility.

### 2.2. Inclusion Criteria

Full-text articles in English that reported outcomes in PE patients who were discharged within 48 h after hospital admission or were not admitted at all and received DOACs were included, with no restrictions on the year of publication. All-cause mortality, PE-related mortality, recurrent VTE, any bleeding, readmission to a medical facility, and readmission to hospital at 30, 60, or 90 days post-discharge were considered as outcomes.

### 2.3. Non-Inclusion Criteria

Studies that did not include PE patients, early-discharged patients, or patients managed as outpatients were excluded. Studies that included such a cohort but managed PE with other anticoagulants were also excluded.

### 2.4. Data Extraction

The datasets included the first author, year of publication, study design, baseline characteristics of the population studied, treatment methods, comorbidities, rates of all-cause mortality, PE-related mortality, recurrent VTE, bleeding events, and readmissions. Data extraction was performed independently by two investigators (A.S.K. and D.V.R.). All disagreements were resolved through adjudication by a third reviewer (E.I.S.).

### 2.5. Risk of Bias Assessment

Two authors (A.S.K. and S.A.M.) independently assessed the quality of the included studies. Any disagreements were resolved either through consensus or adjudication by a third author (I.A.Z.).

The revised Cochrane Risk of Bias assessment tool for randomized trials (RoB 2), available at https://sites.google.com/site/riskofbiastool/welcome/rob-2-0-tool/current-version-of-rob-2 (accessed on 12 August 2024), and the Risk of Bias in Non-Randomized Studies (ROBINS-I) tool, available at https://sites.google.com/site/riskofbiastool/welcome/home/original-2016-version-of-robins-i (accessed on 12 August 2024), were used to evaluate the studies’ methodological quality.

This review is registered in PROSPERO under the registration number CRD42024538602.

### 2.6. Statistical Analysis and Meta-Analysis

For a meta-analysis, random-effects models (DerSimonian–Laird) to assess methodology heterogeneity among included studies were planned to use. I2 statistic was planned to assess statistical heterogeneity. Sensitivity and subgroup analyses were planned to be conducted if data were available. Due to significant heterogeneity in reported data including registered outcomes, patients’ characteristics, and regimens of anticoagulation, it was impossible to conduct a meta-analysis. Data were qualitatively synthesized. Summary and findings are presented using descriptive approach.

## 3. Results

### 3.1. Literature Search

**Figure 1:**
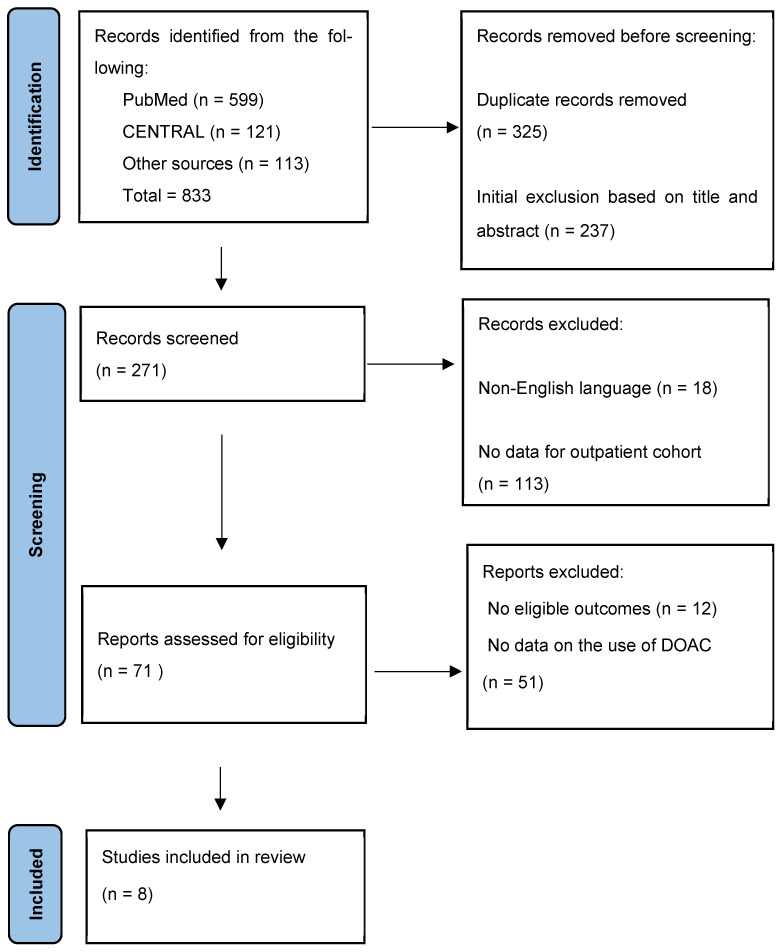
PRISMA flow chart of selection process.

The systematic literature search identified 833 studies. A total of 325 papers were excluded as duplicates, and 237 were excluded as not meeting the inclusion criteria after reviewing their titles and abstracts. Systematic reviews and meta-analyses that were found by searching were also excluded as they did not meet eligibility criteria. Their reference lists were screened for additional publications. Out of the remaining 271 papers, 8 were finally included in this review (Figure 1).

### 3.2. Included Studies

This review included eight studies: two RCTs, four non-RCTs, and two retrospective studies. The main data extracted are summarized in Table 1, Table 2 and Table 3.

Definitions of approach to PE management with no hospital admission varied. In four studies, outpatient management was defined as discharge from the emergency department (ED) within 24 h [12,13,16,18]. In two studies, patients were discharged from the ED immediately [14,17]. In one study, patients were discharged within 48 h [11]. In the study by Chatani et al., patients randomized to the outpatient treatment group were discharged from the emergency department within 12 h [15].

In five studies, only rivaroxaban was used [11,13,14,15,17]. In two studies, some patients received apixaban, edoxaban, or dabigatran. In one study, the majority of patients received apixaban [16]. PE risks were assessed by the Hestia criteria in four studies [11,13,14,17], by the simplified Pulmonary Embolism Severity Index (sPESI) in two studies [12,15], and by both tools in two studies [16,18]. The POMPE-C (Prediction of Mortality from Pulmonary Embolism with Cancer) scale was used in three studies for further stratification of patients with an active malignancy [14,16,17]. Table 2 lists the key demographic characteristics of the patients.

The 30-day rates of all-cause mortality, PE-related mortality, recurrent VTE, and bleeding events were assessed in two studies [16,17], 90-day rates in three studies [11,13,15], and 6-month rates in three studies [12,14,18] (Table 3). Three studies reported readmission to a medical facility [16,17,18]. Three studies reported hospital readmission rates [11,13,15,16,17,18].

### 3.3. Clinical Outcomes

All-cause mortality was low in all studies, independently of the follow-up period. The highest mortality rate reported was 6.1% [15]. However, that study included many cancer patients, which might have led to high overall mortality. None of the papers reported PE-related mortality.

VTE recurrence was remarkably low, with only two studies reporting such cases, with rates of 0.6% and 1.4%. In six studies, no recurrent VTE events were registered.

The rates of any bleeding event varied from 0 to 12.2%. Major bleeding events were rare, with the highest incidence of 4.6% [15].

### 3.4. Risk of Bias

**Figure 2:**
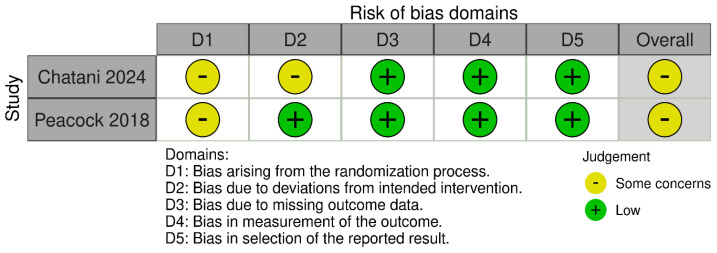
Assessment of the risk of bias in RCTs [13,15].

Both RCTs had a moderate risk of bias (Figure 2) [13,15] due to a lack of detail regarding the randomization process. Deviations from the treatment protocol were found in one of these trials, which may have caused misinterpretation of the outcomes and reduced the internal validity of the findings (Supplementary Materials, Table S3). [15].

**Figure 3:**
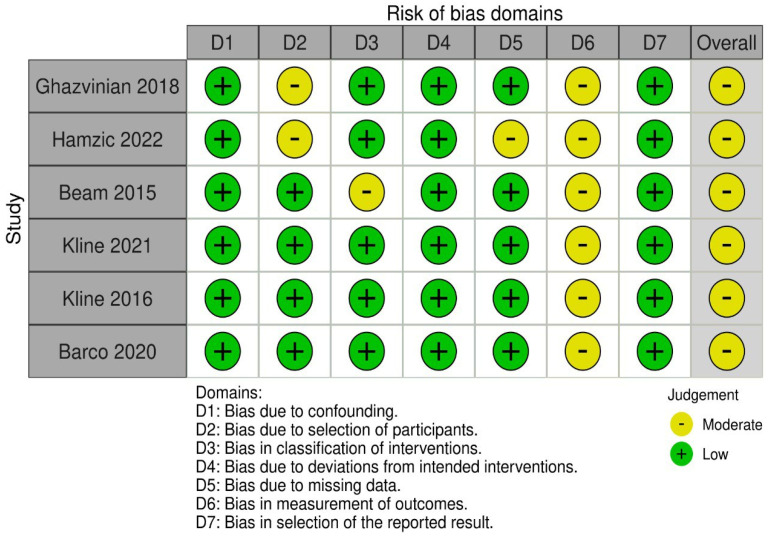
Assessment of the risk of bias in non-RCTs [11,12,14,16,17,18].

All non-RCTs were assessed as having a moderate risk of bias (Figure 3). All studies presented limited data on the blinding of outcome assessors and the interpretation of examination test results [11,12,14,16,17,18]. Uncertainty in participant selection was an issue of concern in two studies [12,18], which may have resulted in deviations in the inclusion criteria. One study was judged as having a risk of bias due to unclear classification of the intervention [14]. Another study was considered to have a moderate risk of bias due to missing data [18], which might have led to incomplete outcome reporting (Supplementary Materials, Table S3).

### 3.5. Meta-Analysis

It was impossible to perform a meta-analysis due to the heterogeneity in study designs, patient selection criteria, risk stratification methods, and therapeutic regimens of the included studies. Consequently, we chose to conduct a qualitative synthesis, providing a descriptive overview of the clinical outcomes and safety endpoints from the articles.

## 4. Discussion

This systematic review aimed to evaluate the safety and efficacy of outpatient treatment of PE patients using DOACs as initial therapy. The previously published systematic review by Maughan et al. confirmed that outpatient PE management could be a beneficial option for both individuals and healthcare systems [19]. The authors discussed management using different types of anticoagulants. They identified only three studies in which DOACs were used for outpatient PE management, including only one RCT. In our review, we focused on the studies in which anticoagulation had started and had been extended with DOACs only. We found eight studies, including one additional RCT by Chatani et al. [15].

We found no papers in which PE was confirmed, and anticoagulation had started and then continued outside the hospital; i.e., in an outpatient clinic. All the patients were initially referred to EDs, in which they were examined, and DOACs were prescribed. In all but one study, patients were discharged within the first 24 h. This approach could be defined as outpatient. The time at the hospital was spent on the patient’s examination and deciding whether the patient had to be admitted or could be managed at home. Simultaneously, there are still no developed and generally accepted formal indications for early discharge. Outpatient management demonstrates a substantial reduction in healthcare costs compared with conventional in-patient treatment [20]. Moreover, many PE patients may prefer to be managed as outpatients as it comforts and allows them to save social connections [13]. Nevertheless, as PE is a potentially life-threatening condition, decisions regarding discharge within several hours after admission can raise medical, legal, and ethical concerns.

All-cause mortality rates appeared to be remarkably low across the studies. Only Chatani et al. [15] reported a rate of 6.1% at 3-month follow-up. We believe this is due to the inclusion of cancer patients in the study. None of the included studies reported PE-related deaths during follow-up. Our findings are similar to those of Maughan et al.’s [19] systematic review, which included studies conducted on patients managed mainly with non-DOACs.

It needs to be considered that PE-related mortality increases with age and among so-called fragile and frail patients [21,22]. The mean age was mainly around 45–60 in the studies included in our review. Chronic lung diseases and CHF were relatively rarely registered among PE patients managed as outpatients, which may indicate that age and comorbidity were the factors that impacted the decision about outpatient management of PE patients. Nevertheless, advanced age per se does not necessitate a prolonged hospital stay, as shown in the HoT-PE trial by Barco et al. [11]. In a subgroup analysis of the HoT-PE cohort, Hobohm et al. [23] assessed the efficacy and safety of early discharge and outpatient treatment of “vulnerable” patients with a PE with a low risk of death. The patients were additionally stratified by age (>75 years), kidney failure (creatinine clearance <50 mL/min), or BMI < 18.5 kg/m^2^. The efficacy and safety were the same in vulnerable and non-vulnerable patients. Major bleeding occurred in three (1.5%) vulnerable vs. three (1%) non-vulnerable patients, respectively. All-cause mortality at three-month follow-up remained low in both groups (0.5% vs. 0.3%, respectively). All the deaths were due to cancer progression.

We found a clear trend to register mortality and major bleeding rates more often in non-randomized trials. Beyond cases reported by Chatani et al. in a cancer patient’s cohort [15], all other all-cause deaths were registered in non-RCTs [11,12,14]. In the other included RCT, this outcome had not been registered [13]. The same tendency was also clear for the rates of VTE recurrence and major bleedings. This outlines the need to conduct more RCTs to obtain robust data regarding outpatient PE management.

Only PE patients with a low risk of death are generally considered candidates for outpatient management. To identify low-risk PE patients, different tools are used. In the included studies four tools were utilized, i.e., Hestia, PESI, sPESI and POMPE-C. This confirms that there is still no consensus on what tool is better and, therefore, should be used as a method of choice. As no PE-related mortality was reported by all included studies, it is impossible to draw any conclusion on this subject. Anyway, detailed analysis allowed us to say that a better assessment score has to be selected or developed and studied in the future. This is confirmed by questionable results of sPESI utilized as a screening tool. The most often used tool was the Hestia rule, which had been developed specifically for identifying those who are ineligible for home PE treatment. On the one hand, in the HOME-PE randomized trial, sPESI was confirmed as a tool that can be used for selecting patients for outpatient management [24]. This was also shown in the meta-analysis conducted by Palas et al., who calculated the risk of misclassification of high-risk patients in low-risk as 5 (95% CI: 3–11) for Hestia and 2 (95% CI: 1–6) for sPESI, for every 1000 patients with PE in terms of mortality [25]. At the same time, Roy PM et al. discussed disadvantages of sPESI classification as patients consider a high-risk even if their age is 80> or cancer is present without taking other factors into account [24]. Thus, a hemodynamically stable patient has to be managed in a hospital setting as having high risk of death just because of age or any cancer. That is, sPESI should not be applied as a standalone rule for deciding if a PE patient is eligible for outpatient management was also confirmed by two studies included in our review. Barco et al. adapted the inclusion criteria to the Hestia rule [11]. After calculating sPESI in included patients, they found that the score was ≥1 in 107 cases thus stratifying patients as high-risk [11]. At the same time, of the three cases of PE recurrence, only one developed in a patient with sPESI ≥ 1. Among the six major bleeding cases, only two developed in patients who had sPESI ≥ 1. Chatani et al. recruited PE patients who had a cancer at inclusion [15]. Thus, these study patients had sPESI 1 and more, which would have led to ineligibility for home treatment in a real-life practice. But no PE-related deaths or even VTE recurrences were registered over a 3-month follow-up in included patients. We believe that sPESI should not be used for stratifying eligibility of PE patients for outpatient management. At least, sPESI should not be the only tool clinical decisions should be based.

While our findings confirm that outpatient PE management in selected cases is a promising approach, it has to be taken into account that body of evidence is still not that sufficient. Despite the fact that at least two RCTs were conducted on the DOAC usage for home treatment of PE adopting GRADE system (Grading of Recommendations, Assessment, Development, and Evaluations) principles, it can be judged that an outpatient approach can only be suggested with a low level of evidence due to heterogeneity of study designs, patient groups, risk assessment tools, DOAC types, and outcome definitions.

### Limitations

This systematic review has several limitations. Most included studies were observational with a moderate risk of bias, limiting the strength of conclusions. There was substantial heterogeneity in study designs, patient selection, outcomes, and follow-up durations, which prevented a quantitative meta-analysis. The use of different scores and rules for PE patients’ selection for outpatient management is also a limitation. Not all the rules are suitable for the selection, and at least sPESI should be used for this purpose with caution. Future studies are needed for establishing a better tool for eligibility assessment for outpatient PE management. Different direct oral anticoagulants (DOACs) were used, including rivaroxaban, apixaban, dabigatran, and edoxaban, with variations in dosing and treatment protocols, which may affect both efficacy and safety. Another limitation is the significant difference in the number of cancer patients in included studies. Taking into account that this cohort is under the elevated risks of both VTE recurrence and bleeding due to anticoagulation therapy, there should be caution in analyzing data on outpatient PE management results. There is a need for studies designed on different patients’ cohorts, i.e., with and without cancer. Some studies reported incomplete or inconsistent outcome data, further limiting generalizability. Therefore, the conclusions regarding the efficacy and safety of outpatient treatment of pulmonary embolism with DOACs should be considered preliminary and must be confirmed in large-scale RCTs.

## 5. Conclusions

Outpatient management of PE with DOACs may be beneficial for selected patients. For the selection of patients, Hestia, POMPE-C and RESI scores and rules can be used, but utilizing sPESI as the only tool has to be avoided. The available data confirm low rates of all-cause and PE-related mortality as well as VTE recurrence. DOACs seem to be safe, as rates of bleeding events—including major ones—are low. However, the heterogeneity in the published data indicates a need for larger prospective studies to establish the role of DOAC-based outpatient management of PE, particularly in elderly, fragile, and frail patients.

## Figures and Tables

**Table 1:** Studies selected for systematic review.

Reference	Design	Country	Patients with PE (*n*)	Anticoagulants Used	Approach (Early Discharge; Outpatient)	Assessment Tool for Risk of Death
Barco et al. [11]	Non-RCT	Germany	525	Rivaroxaban	Discharged within 48 h after hospital admission	Hestia ^1^
Ghazvinian et al. [12]	Non-RCT	Sweden	245	Rivaroxaban—225 Apixaban—23 Dabigatran—2 ^3^	Discharged within 24 h after hospital admission	sPESI
Peacock et al. [13]	RCT	USA	51	Rivaroxaban	Discharged within 24 h after hospital admission	Hestia
Beam et al. [14]	Non-RCT	USA	35	Rivaroxaban	Immediate discharge from ED (no time specified)	Hestia; POMPE -C
Chatani et al. [15]	RCT	Japan	66	Rivaroxaban	Discharged from ED (<12 h)	sPESI
Kline et al. [16]	Non-RCT	USA	518	Rivaroxaban; apixaban ^2^ (shares in PE patients were not reported)	Discharged within 24 h after hospital admission	Hestia; sPESI; POMPE -C
Kline et al. [17]	Non-RCT	USA	67	Rivaroxaban	Immediate discharge from ED (no time specified)	Hestia; POMPE -C
Hamzić et al. [18]	Non-RCT	Croatia	42	Rivaroxaban—33 Apixaban—4 Edoxaban—1	Discharged within 24 h after hospital admission	Hestia; PESI; sPESI

^1^ Non-inclusion criteria were adapted to the Hestia criteria. ^2^ Exact numbers of PE patients who received rivaroxaban or apixaban were not reported. ^3^ Some patients switched from one DOAC to another.

**Table 2:** Basic characteristics of patients.

Reference	Mean Age (SD)	Female, n (%)	Risk of Death	Inherited Thrombophilia, n (%)	Provoked PE, n (%)	Concomitant DVT, n (%)	Cancer, n (%)	History of VTE, n (%) ^1^	Chronic Lung Disease, n (%)	CHF ^2^, n (%)
Barco et al. [11]	56.7 (NA)	240 (45.7)	Low	NA	NA	214 (40.8)	32 (6.2)	PE—39 (7.5) DVT—82 (15.9)	26 (5)	7 (1.3)
Ghazvinian et al. [12]	60 (17.2)	120 (49)	Low	29 (12)	NA	3 (1)	14 (6)	DVT—3 (1)	11 (5)	27 (11)
Peacock et al. [13]	49.14 (13.3)	27 (52.9)	Low	NA	NA	NA	3 (5.9)	PE—8 (15.7) DVT—2 (3.9)	NA	1 (2)
Beam et al. [14]	50 (NA)	NA ^3^	Low	NA	NA	5 (14)	5 (4.7)	NA	NA	1 (0.9)
Chatani et al. [15]	66.2 (9.5)	34 (52)	High	NA	NA	40 (61)	28 (42)	6 (9.1)	NA	NA
Kline et al. [16]	(NA)	254 (49)	Low	NA	NA	38 (7.3)	Active—16 (3) Remission—25 (5)	326 (63)	40 (8)	19 (4)
Kline et al. [17]	41.5 (NA)	NA ^3^	Low	NA	NA	7 (10)	NA	NA	NA	NA
Hamzić et al. [18]	54 (NA)	24 (57.1)	14 (33.3%)—high	NA	NA	Proximal—2 (4.8) Distal—11 (26)	12 (28.6)	PE—8 (19) DVT—9 (21.4)	NA	6 (14.3)

^1^ A history of any prior event of deep vein thrombosis (DVT) or PE. ^2^ Chronic heart failure (CHF). ^3^ Gender distribution among PE patients was not reported. NA—not available.

**Table 3:** Mortality, recurrent VTE, and bleeding rates.

Reference	Follow-Up	Participants, (n)	All-Cause Mortality, n (%)	PE-Caused Mortality, n (%)	Recurrent VTE, n (%)	Any Bleeding, n (%)	Major Bleeding, n (%)	Clinically Relevant Non-Major Bleeding, n (%)	Minor Bleeding, n (%)
Barco et al. [11]	3 m	525	2 (0.4)	0	3 (0.6)	37 (7.2)	6 (1.2)	31 (6)	0
Ghazvinian et al. [12]	6 m	245	1 (0.4)	0	0	1 (0.4)	0	1 (0.4)	5 (2)
Peacock et al. [13]	3 m	51	0	0	0	1 (2)	0	1 (2)	0
Beam et al. [14]	6 m	35	2 (1.9)	0	0	NA	NA	NA	NA
Chatani et al. [15]	3 m	66	4 (6.1)	0	0	6 (9.2)	3 (4.6)	3 (4.6)	0
Kline et al. [16]	30 d	518	0	0	7 (1.4)	52/425 (12.2) ^1^	1(0.2)	4 (0.8)	NA
Kline et al. [17]	30 d	67	0	0	0	N ^2^	0	NA ^2^	NA ^2^
Hamzić et al. [18]	6 m	42	0	0	0	0	0	0	0

^1^ The data reported on 425 patients who were available for the contact. ^2^ No details on bleeding reported, except that none of the PE patients had major bleeding during follow-up.

## Data Availability

No new data were created or analyzed in this review. Data sharing is inapplicable to this paper.

## Supplementary Materials

The following supporting information can be downloaded at: https://www.mdpi.com/article/10.3390/jcm14248931/s1, Table S1: PRISMA 2020 Main Checklist; Table S2: PRISMA Abstract Checklist. Table S3: Risk of bias in the studies included in this review [26].
