# Supplementary material for: Outpatient Management of Pulmonary Embolism Patients with Direct Oral Anticoagulants: A Systematic Review
Source: J Clin Med. 2025 Dec 17;14(24):8931. doi: 10.3390/jcm14248931 (PMC12734285; doi:10.3390/jcm14248931)
Supplement: Supplementary file 1 [file jcm-14-08931-s001.zip › jcm-4012284-supplementary.pdf]

Table S1:PRISMA 2020 Main Checklist

Topic	No.	Item	Location where item is reported
**TITLE**			
**Title**	1	Identify the report as a systematic review.	Section "Title", Page 1
**ABSTRACT**			
**Abstract**	2	See the PRISMA 2020 for Abstracts checklist	
**INTRODUCTION**			
**Rationale**	3	Describe the rationale for the review in the context of existing knowledge.	Section "Introduction", Line 48-65
**Objectives**	4	Provide an explicit statement of the objective(s) or question(s) the review addresses.	Section "Introduction", Line 66-67
**METHODS**			
**Eligibility criteria**	5	Specify the inclusion and exclusion criteria for the review and how studies were grouped for the syntheses.	Section "Methods", Line 112-117, Line 119-121
**Information sources**	6	Specify all databases, registers, websites, organisations, reference lists and other sources searched or consulted to identify studies. Specify the date when each source was last searched or consulted.	Section "Methods", Line 70-77
**Search strategy**	7	Present the full search strategies for all databases, registers and websites, including any filters and limits used.	Section "Methods", Line 78-107
**Selection process**	8	Specify the methods used to decide whether a study met the inclusion criteria of the review, including how many reviewers screened each record and each report retrieved, whether they worked independently, and if applicable, details of automation tools used in the process.	Item is not reported
**Data collection process**	9	Specify the methods used to collect data from reports, including how many reviewers collected data from each report, whether they worked independently, any processes for obtaining or confirming data from study investigators, and if applicable, details of automation tools used in the process.	Section "Methods", Line 125-127

Topic	No.	Item	Location where item is reported
**Data items**	10a	List and define all outcomes for which data were sought. Specify whether all results that were compatible with each outcome domain in each study were sought (e.g. for all measures, time points, analyses), and if not, the methods used to decide which results to collect.	Section "Methods", Line 123-125
	10b	List and define all other variables for which data were sought (e.g. participant and intervention characteristics, funding sources). Describe any assumptions made about any missing or unclear information.	Item is not reported
**Study risk of bias assessment**	11	Specify the methods used to assess risk of bias in the included studies, including details of the tool(s) used, how many reviewers assessed each study and whether they worked independently, and if applicable, details of automation tools used in the process.	Section "Methods", Line 129-137
**Effect measures**	12	Specify for each outcome the effect measure(s) (e.g. risk ratio, mean difference) used in the synthesis or presentation of results.	Item is not reported
**Synthesis methods**	13a	Describe the processes used to decide which studies were eligible for each synthesis (e.g. tabulating the study intervention characteristics and comparing against the planned groups for each synthesis (item 5)).	Item is not reported
	13b	Describe any methods required to prepare the data for presentation or synthesis, such as handling of missing summary statistics, or data conversions.	Item is not reported
	13c	Describe any methods used to tabulate or visually display results of individual studies and syntheses.	Section "Methods", Line 147
	13d	Describe any methods used to synthesize results and provide a rationale for the choice(s). If meta-analysis was performed, describe the model(s), method(s) to identify the presence and extent of statistical heterogeneity, and software package(s) used.	Section "Methods", Line 146-147
	13e	Describe any methods used to explore possible causes of heterogeneity among study results (e.g. subgroup analysis, meta-regression).	Section "Methods", Line 142-146
	13f	Describe any sensitivity analyses conducted to assess robustness of the synthesized results.	Item is not reported
**Reporting bias assessment**	14	Describe any methods used to assess risk of bias due to missing results in a synthesis (arising from reporting biases).	Item is not reported

Topic	No.	Item	Location where item is reported
**Certainty assessment**	15	Describe any methods used to assess certainty (or confidence) in the body of evidence for an outcome.	Item is not reported
**RESULTS**			
**Study selection**	16a	Describe the results of the search and selection process, from the number of records identified in the search to the number of studies included in the review, ideally using a flow diagram.	Section "Results", Line 150-155
	16b	Cite studies that might appear to meet the inclusion criteria, but which were excluded, and explain why they were excluded.	Item is not reported
**Study characteristics**	17	Cite each included study and present its characteristics.	Section "Results", Line 222-243
**Risk of bias in studies**	18	Present assessments of risk of bias for each included study.	Section "Results", Line 272-284
**Results of individual studies**	19	For all outcomes, present, for each study: (a) summary statistics for each group (where appropriate) and (b) an effect estimate and its precision (e.g. confidence/credible interval), ideally using structured tables or plots.	Table 3.
**Results of syntheses**	20a	For each synthesis, briefly summarise the characteristics and risk of bias among contributing studies.	Supplement 1.
	20b	Present results of all statistical syntheses conducted. If meta-analysis was done, present for each the summary estimate and its precision (e.g. confidence/credible interval) and measures of statistical heterogeneity. If comparing groups, describe the direction of the effect.	Item is not reported
	20c	Present results of all investigations of possible causes of heterogeneity among study results.	Item is not reported
	20d	Present results of all sensitivity analyses conducted to assess the robustness of the synthesized results.	Item is not reported
**Reporting biases**	21	Present assessments of risk of bias due to missing results (arising from reporting biases) for each synthesis assessed.	Item is not reported
**Certainty of evidence**	22	Present assessments of certainty (or confidence) in the body of evidence for each outcome assessed.	Item is not reported
**DISCUSSION**			

Topic	No.	Item	Location where item is reported
**Discussion**	23a	Provide a general interpretation of the results in the context of other evidence.	Section "Discussion", Line 293-374
	23b	Discuss any limitations of the evidence included in the review.	Section "Discussion", Line 376-393
	23c	Discuss any limitations of the review processes used.	Item is not reported
	23d	Discuss implications of the results for practice, policy, and future research.	Section "Discussion", Line 391-393; Section "Conclusion", Line 395-399
**OTHER INFORMATION**			
**Registration and protocol**	24a	Provide registration information for the review, including register name and registration number, or state that the review was not registered.	Section "Methods", Line 138-139
	24b	Indicate where the review protocol can be accessed, or state that a protocol was not prepared.	Item is not reported
	24c	Describe and explain any amendments to information provided at registration or in the protocol.	Item is not reported
**Support**	25	Describe sources of financial or non-financial support for the review, and the role of the funders or sponsors in the review.	Section "Financial support statement", Line 404-405
**Competing interests**	26	Declare any competing interests of review authors.	Section "Conflict of Interests", Line 419-420
**Availability of data, code and other materials**	27	Report which of the following are publicly available and where they can be found: template data collection forms; data extracted from included studies; data used for all analyses; analytic code; any other materials used in the review.	Section "Data availability statement", Line 417-418

Table S2: PRIMSA Abstract Checklist

Topic	No.	Item	Reported?
**TITLE**			
**Title**	1	Identify the report as a systematic review.	Yes
**BACKGROUND**			
**Objectives**	2	Provide an explicit statement of the main objective(s) or question(s) the review addresses.	Yes
**METHODS**			
**Eligibility criteria**	3	Specify the inclusion and exclusion criteria for the review.	Yes
**Information sources**	4	Specify the information sources (e.g. databases, registers) used to identify studies and the date when each was last searched.	Yes
**Risk of bias**	5	Specify the methods used to assess risk of bias in the included studies.	No
**Synthesis of results**	6	Specify the methods used to present and synthesize results.	No
**RESULTS**			
**Included studies**	7	Give the total number of included studies and participants and summarise relevant characteristics of studies.	Yes
**Synthesis of results**	8	Present results for main outcomes, preferably indicating the number of included studies and participants for each. If meta-analysis was done, report the summary estimate and confidence/credible interval. If comparing groups, indicate the direction of the effect (i.e. which group is favoured).	Yes
**DISCUSSION**			
**Limitations of evidence**	9	Provide a brief summary of the limitations of the evidence included in the review (e.g. study risk of bias, inconsistency and imprecision).	No
**Interpretation**	10	Provide a general interpretation of the results and important implications.	No
**OTHER**			
**Funding**	11	Specify the primary source of funding for the review.	No
**Registration**	12	Provide the register name and registration number.	No

From: Page MJ, McKenzie JE, Bossuyt PM, Boutron I, Hoffmann TC, Mulrow CD, et al. The PRISMA 2020 statement: an updated guideline for reporting systematic reviews. MetaArXiv. 2020, September 14. DOI: 10.31222/osf.io/v7gm2. For more information, visit: www.prisma-statement.org[26]

Table **S3. Risk of bias in the studies included in the review**

Chatani et al. 2024 [15] risk of bias

Bias	*Authors' judgement*	*Support for judgement*
Bias arising from the randomization process	*Some concerns*	*«...patients were not randomized into home treatment; selection of home treatment or in-hospital treatment was left to the discretion of the attending physician...»*
Bias due to deviations from intended intervention	*Some concerns*	*«...premature discontinuation of rivaroxaban within 3 months was similar between the groups (9.1% vs. 9.8%, P=1.00).»*
Bias due to missing outcome data	*Low risk*	*«All patients were followed for 3 month...»* *«After excluding 1 patient who withdrew consent during the follow-up period within 3 months, 178 patients were included in the current 3-month analysis.»*
Bias in measurement of the outcome	*Low risk*	*«...an independent data and safety monitoring committee...adjudicator-blinded RCT...»*
Bias in selection of the reported result	*Low risk*	*«This predetermined companion report...was predetermined in the protocol before the start... as an exploratory type of subanalysis.»*

Peacock et al. 2018 [13] risk of bias

Bias	*Authors' judgement*	*Support for judgement*
Bias arising from the randomization process	*Some concerns*	*«Adult subjects were randomized to early ED discharge on rivaroxaban or SOC»; «...open-label randomized trial...»*
Bias due to deviations from intended intervention	*Low risk*	*«Patients randomized to early discharge on rivaroxaban...were instructed to take rivaroxaban ... 15 mg twice daily for 21 days and then 20 mg once daily...»*
Bias due to missing outcome data	*Low risk*	*«A total of 114 subjects were randomized and 99 (86.8%) completed the study.»*
Bias in measurement of the outcome	*Low risk*	*«A 90-day composite safety endpoint was defined as major bleeding, clinically relevant nonmajor bleeding, and mortality.»*
Bias in selection of the reported result	*Low risk*	*«Of 112 (98.2%) receiving at least one dose of study drug, 99 (86.8%) completed the study»; « At 90 days, there were no bleeding events, recurrent VTE, or deaths.»*

Ghazvinian et al. 2018 [12] risk of bias

Bias	*Authors' judgement*	*Support for judgement*
Bias due to confounding	*Low risk*	*«The selection of patients, both for DOAC instead for warfarin treatment, and for outpatient instead of inpatient treatment of course constitutes another limitation of our study.»*
Bias due to selection of participants	*Some concerns*	*«...245 of the 881 (28%) patients ...had been selected for outpatient treatment,*

		i.e., DOAC treatment had been started already during an emergency department (ED) visit not exceeding 24 h.»
Bias in classification of interventions	*Low risk*	*«We extracted data from AuriculA.... for all 881...treated with DOAC for PE... during 2013–2015...»*
Bias due to deviations from intended interventions	*Low risk*	*No details provided.*
Bias due to missing data	*Low risk*	*«...files and imaging data for all 881 patients were hereafter reviewed by the authors.»*
Bias in measurement of outcomes	*Some concerns*	*«...one patient died; a 72 years old male patient with cardiac arrest of unknown cause...the patient's relatives declined autopsy.»*
Bias in selection of the reported result	*Low risk*	*«The major limitations of the study are its retrospective nature, and the lack of randomization.»*

Hamzić et al. 2022 [18] risk of bias

Bias	*Authors' judgement*	*Support for judgement*
Bias due to confounding	*Low risk*	*«The risk assessment tools - HESTIA score, PESI score, sPESI score, and the VTE-BLEED bleeding risk assessment tool - were also retrospectively analyzed».*
Bias due to selection of participants	*Some concerns*	*«The total number of patients diagnosed with pulmonary embolism in the two years was 457. 9.2% of patients were treated in outpatient settings.»*
Bias in classification of interventions	*Low risk*	*«42 patients discharged after a short-term observation from the emergency department (<24h)... Ninety-one*

		percent…discharged with direct oral anticoagulant…»
Bias due to deviations from intended interventions	*Low risk*	*«More than 90% of patients were discharged… with DOAC therapy, in most cases with rivaroxaban.»;*
Bias due to missing data	*Some concerns*	*«…we…analyzed…potential complications…up to 6 months after discharge.»*
Bias in measurement of outcomes	*Some concerns*	*«We did not notice any adverse events (hemorrhage, progression of PE, or major cardiovascular issues).»*
Bias in selection of the reported result	*Low risk*	*«There were no cases of thrombosis progression, bleeding, or major cardiovascular events.»*

Beam et al. 2015 [14] risk of bias

Bias	*Authors' judgement*	*Support for judgement*
Bias due to confounding	*Low risk*	*«Patients were determined to be low-risk by using a modified version of the Hestia criteria, supplemented by additional criteria for patients with active cancer.»*
Bias due to selection of participants	*Low risk*	*«…27% of the PEs and 51% of DVTs diagnosed in the ED were discharged from the ED on rivaroxaban…»*
Bias in classification of interventions	*Some concerns*	*«A protocol was established for treating low-risk DVT or PE patients with rivaroxaban… patients were prescribed 15 mg of rivaroxaban twice per day for 21 days…»*
Bias due to deviations from intended interventions	*Low risk*	*«One to 2 days after discharge, a member of the care team called the patient to confirm that the patient was able to fill.»*

Bias due to missing data	*Low risk*	*«Three patients were lost to follow-up...»; «For patients who could not be contacted by telephone, we examined a comprehensive electronic medical record system (the Indiana Network for Patient Care [INPC]...for any outcome measure.»*
Bias in measurement of outcomes	*Some concerns*	*«Outcomes were determined through patient follow-up visits, telephone conversations, and chart extraction.»*
Bias in selection of the reported result	*Low risk*	*«None of 106 patients... developed VTE recurrence on therapy. While taking anticoagulation, none of the 106... patients experienced clinically significant bleeding... three of 106 patients... experienced VTE recurrence within 1 year after discontinuation of anticoagulation.»*

Kline et al. 2021 [16] risk of bias

Bias	*Authors' judgement*	*Support for judgement*
Bias due to confounding	*Low risk*	*«Participants had newly diagnosed VTE with low risk of death based upon either the modified Hestia criteria, or physician judgment plus the simplified PE severity index score of zero, together with non-high bleeding risk...»*
Bias due to selection of participants	*Low risk*	*«This was a single-arm trial, conducted... in 33 EDs...»*
Bias in classification of interventions	*Low risk*	*Patients had to be discharged within 24 hours of triage and treated with*

		either apixaban or rivaroxaban.
Bias due to deviations from intended interventions	*Low risk*	*«Medication non-adherence was reported by patients in 8.0% (6.6–9.5%), and was associated with a risk ratio… for VTE recurrence.»*
Bias due to missing data	*Low risk*	*«We enrolled 1421 patients with complete outcomes data… no patient died… serious adverse events occurred in 2.5%...»*
Bias in measurement of outcomes	*Some concerns*	*«Effectiveness was defined by the primary efficacy and safety outcomes, image-proven recurrent VTE and bleeding requiring hospitalization >24 hours…»*
Bias in selection of the reported result	*Low risk*	*«The recurrent VTE requiring hospitalization occurred in 14/1421…and bleeding requiring hospitalization occurred in 12/1421…»*

Kline et al. 2016 [17] risk of bias

Bias	*Authors' judgement*	*Support for judgement*
Bias due to confounding	*Low risk*	*«…a protocol that selected VTE patients as low-risk patients by the Hestia criteria, and initiated home anticoagulation with an oral factor Xa antagonist.»*
Bias due to selection of participants	*Low risk*	*«From April 2013 to September 2015, 253 patients were treated, including 67 with PE.»*
Bias in classification of interventions	*Low risk*	*«…patients… initiated home anticoagulation with an oral factor Xa antagonist.»*
Bias due to deviations from intended interventions	*Low risk*	*«Patients…with…a follow-up visit scheduled at 3–6 months*

Bias due to missing data	*Low risk*	*«...structured collection form... supplemented by telephone calls by a research coordinator...medical records were also reviewed...The coordinator used explicit definitions...A 10% random sample...interobserver variability ...100% agreement.»*
Bias in measurement of outcomes	*Some concerns*	*«The primary outcomes were VTE recurrence and hemorrhage at 30 days.»*
Bias in selection of the reported result	*Low risk*	*«Within 30 days, 2/253 patients had recurrent DVT and 2/253 had major hemorrhage...»*

Barco et al. 2020 [11] risk of bias

Bias	*Authors' judgement*	*Support for judgement*
Bias due to confounding	*Low risk*	*«Eligibility criteria included absence of (i) haemodynamic instability, (ii) right ventricular dysfunction...and (iii) serious comorbidities.»*
Bias due to selection of participants	*Low risk*	*«From May 2014 through June 2018, consecutive patients were enrolled in seven countries.»*
Bias in classification of interventions	*Low risk*	*«Rivaroxaban was given at the approved dose for PE for ≥3 months.»*
Bias due to deviations from intended interventions	*Low risk*	*«Up to two nights of hospital stay were permitted. »*
Bias due to missing data	*Low risk*	*«All efficacy and safety outcomes were adjudicated by an independent clinical events committee...»*
Bias in measurement of outcomes	*Some concerns*	*«The primary efficacy outcome was symptomatic*

		recurrent VTE, or PE-related death within 3 months of enrolment.»
Bias in selection of the reported result	*Low risk*	*«Of the 525 patients…three (0.6) suffered symptomatic non-fatal VTE recurrence…Major bleeding occurred in 6 (1.2%)…»*
